# *Actinomyces israelii* and *Fusobacterium nucleatum* brain abscess in an immunocompetent patient: case report

**DOI:** 10.1099/acmi.0.000499.v4

**Published:** 2023-06-26

**Authors:** Mouhsine Lamtri Laarif, Raphael Schils, Fréderic Lifrange, Christophe Valkenborgh, Pauline Pitti, Pauline Brouwers, Elettra Bianchi, Cécile Meex, Marie-Pierre Hayette

**Affiliations:** ^1^​ Department of Clinical Biology, Microbiology, University of Liège Hospital, Liège, Belgium; ^2^​ Department of Internal Medicine, Infectious Diseases, University of Liège Hospital, Liège, Belgium; ^3^​ Department of Pathology, University of Liege Hospital, Liège, Belgium; ^4^​ Department of Radiology, University of Liege Hospital, Liège, Belgium

**Keywords:** *Actinomyces israelii*, actinomycosis, brain abscesses, *Fusobacterium nucleatum*, periodontitis

## Abstract

**Introduction.:**

Brain abscess is the most common focal infectious neurological injury. Until the nineteenth century this condition was fatal, however the development of neuroimaging for early diagnosis, neurosurgery and antibiotic therapy in the twentieth century has led to new therapeutic strategies decreasing mortality from 50 % in the 1970s to less than 10 % nowadays. In this context we report a case of brain abscess with a dental origin.

**Case report.:**

A immunocompetent man without any addiction presented to the emergency department with dysarthria and frontal headache at home. The clinical examination was normal. Further investigations revealed a polymicrobial brain abscess as a consequence of an ear, nose or throat (ENT) infection with locoregional extension with a dental starting point involving *Actinomyces israelii* and *Fusobacterium nucleatum *. In spite of a rapid diagnosis and a neurosurgical management associated with an optimal treatment by a dual therapy made of ceftriaxone and metronidazole the patient unfortunately died.

**Conclusion.:**

This case report shows that despite a low incidence and a good prognosis following the diagnosis, brain abscesses can lead to patient’s death. Thereby, when the patient’s condition and urgency allow, a thorough dental examination of patients with neurological signs following the recommendations would improve the diagnosis made by the clinician. The use of microbiological documentation, the respect of pre-analytical conditions, the interaction between the laboratory and the clinicians are indispensable for an optimal management of these pathologies.

## Data summary

In order to carry out this work, we have associated all the services involved in the diagnosis and management of the patient with the publication. The clinical data were retrieved from the patient’s medical record via the OMNIPRO software. The magnetic resonance imaging and X-rays were transmitted and interpreted by the radiology department. Grocott and hematoxylin and eosin stains were performed and interpreted by the anatomopathology department. Gram-staining and bacterial culture results were provided and interpreted by the microbiology department. The therapeutic strategy and antibiotic therapy was passed on and decided by the infectious diseases department.

In this clinical case all the examinations that were carried out were described in detail. No additional data needed to be added.

## Introduction

Brain abscess is an intracerebral infection characterized by localized cerebritis that develops into a focus of pus, bounded by a well-vascularized capsule, in response to infection or trauma [1]. Pyogenic infections of the central nervous system (CNS) may be a consequence of traumatic or surgical events (10–20 %), bacterial invasion of the brain via neighbouring sites (20–30 %) or by hematogenous spread (20–40 %) [2, 3]. The incidence of brain abscesses is estimated to one to eight cases per 100 000 population per year [3, 4] with a masculine predominance [5–7]. Improvements in neuroimaging, neurosurgery and antibiotic therapy have led to new therapeutic strategies that have reduced mortality from 50 % in the 1970s to less than 10 % nowadays [3, 4]. Although the most common distant site origins of brain abscess are pulmonary or cardiac [8], a few rare clinical cases have been reported in the literature with a dental origin such as caries, gingivitis, periodontitis, or osteomyelitis of the jaws [6, 9, 10].

We describe here a fatal case of an immunocompetent patient with probable periodontitis causing *Actinomyces israelii (A. israelii*) and *Fusobacteruium nucleatum (F. nucleatum*) brain abscess leading to the patient’s death in spite of an adapted antibiotic therapy.

## Case presentation

### Investigations

An immunocompetent man presented spontaneously to the emergency department with several episodes of intermittent dysarthria, which have been evolving since a week, followed by attention and language disorders. The patient also suffered from frontal headaches responding to level I analgesics and a progressive decrease in visual acuity, which has been evolving for several weeks. He was not addicted to any drugs.

### Clinical findings

**Fig. 1.**
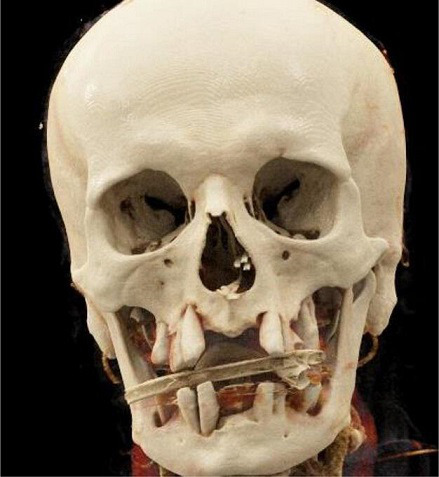
Volume Rendering Technique of cranial CT scan demonstrating alveolar bone loss and significant dental degradation.

During his admission, his parameters were normal except for a *de novo* hypertension of 151/97 mmHg. Also, the general clinical and oral examination of the patient, revealed only significant dental degradation (Fig. 1) and a significant loosening of the teeth 25/26/37 due to his very poor dental hygiene. The neurological examination showed a left lateral homonymous hemianopia and dysarthria. Blood tests showed a mild inflammatory syndrome with c-reactive protein at 14 mg l^−1^ (normal range : <5 mg l^−1^), fibrinogen at 4.8 g l^−1^ (normal range: 1.79–3.86 g l^−1^), white blood cells at the upper limit of the reference range: 8.60×10^3^ /mm^3^ (normal range: 4.60×10^3^–10.10×10^3^ /mm^3^), and mild hyponatremia 135 mmol l^−1^ (normal range: 136–145 mmol l^−1^). Blood cultures taken at the admission were negative after 5 days of incubation. A brain computed tomography (CT) showed an intracranial mass syndrome (49×41×35 mm) in the right parieto-temporo-occipital junction.

**Fig. 2.**
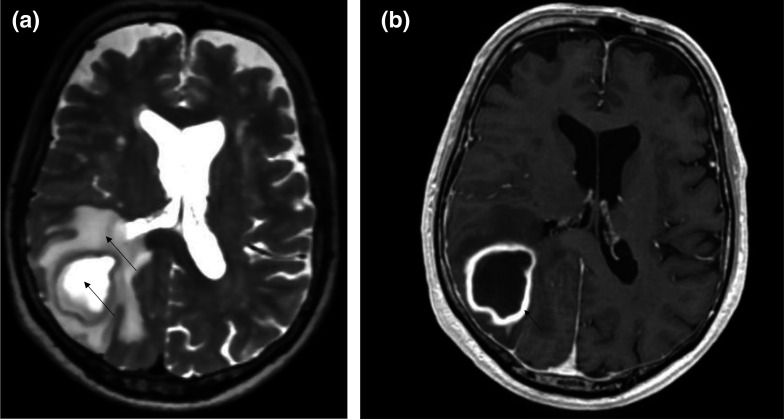
(**a**): Axial section in T2 sequence showing hypo-isointense content and a significant oedema around the mass. (**b**) Axial slice T1sequence +contrast showing a mass with regular contours and homogeneous wall enhancement.

**Fig. 3.**
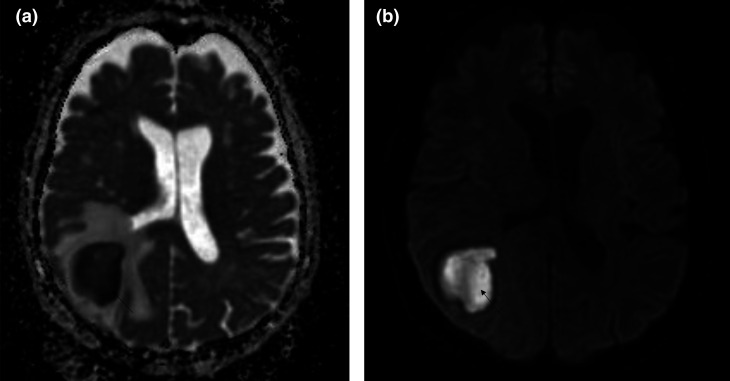
Restriction of diffusion at the mass level and lack of restriction at the capsule level argues for an infectious process. (**a**): Apparent Diffusion Coefficent (ADC) sequence (**b**): Diffusion-weighted imaging (DWI) sequence.

When the patient was hospitalized, brain magnetic resonance imaging (MRI) showed a lesion with hyper-/isointense-T2 fluid content and diffusion-restricting (Fig. 2a). The walls of the lesion appeared relatively regular and homogeneously enhanced (Fig. 2b), allowing us to conclude, as a first hypothesis in the presence of an abscess of the right posterior junction (Fig. 3). The thoracic-abdominal-pelvic CT scan refuted the presence of a primary oncological or infectious lesion.

### Diagnostic assessments

Based on various arguments in favour of the initial hypothesis, the patient was admitted in neurosurgery for drainage of this abscess in the right temporo-parietal-occipital junction.

### Therapeutic interventions

Despite the suspicion of a brain abscess, but given the absence of clinical evidence of sepsis (according to quick sequential organ failure assessment criteria), no antibiotic therapy was started pre-operatively.

**Fig. 4.**
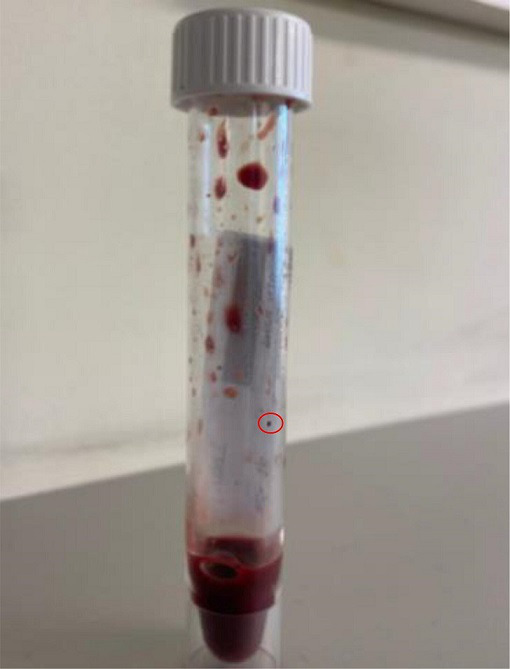
Collection of brain abscess showing sulphur granules.

During surgery, the cerebrospinal fluid was drained, and no intraoperative complication was observed. The diagnosis of brain abscess was confirmed by the collection of a thick purulent fluid in the the right temporoparietal-occipital junction (Fig. 4). The abscess fluid was subsequently sent to the laboratory for bacteriological and histological analysis.

Post-operatively, the patient rapidly developed a septic state with diaphoresis, acute confusion, fever at 39.6 °C and tachypnea (30 min^−1^). In this context, empirical antibiotics were started after taking two blood cultures from different anatomical sites, including ceftriaxone IV 2 g once daily, metronidazole 1500 mg once daily and vancomycin IV 40 mg/kg/24 h. The neurological state deteriorated rapidly with the occurrence of left hemiplegia associated with an altered state of consciousness (Glasgow score 12/15), which led to admission to intensive care.

**Fig. 5.**
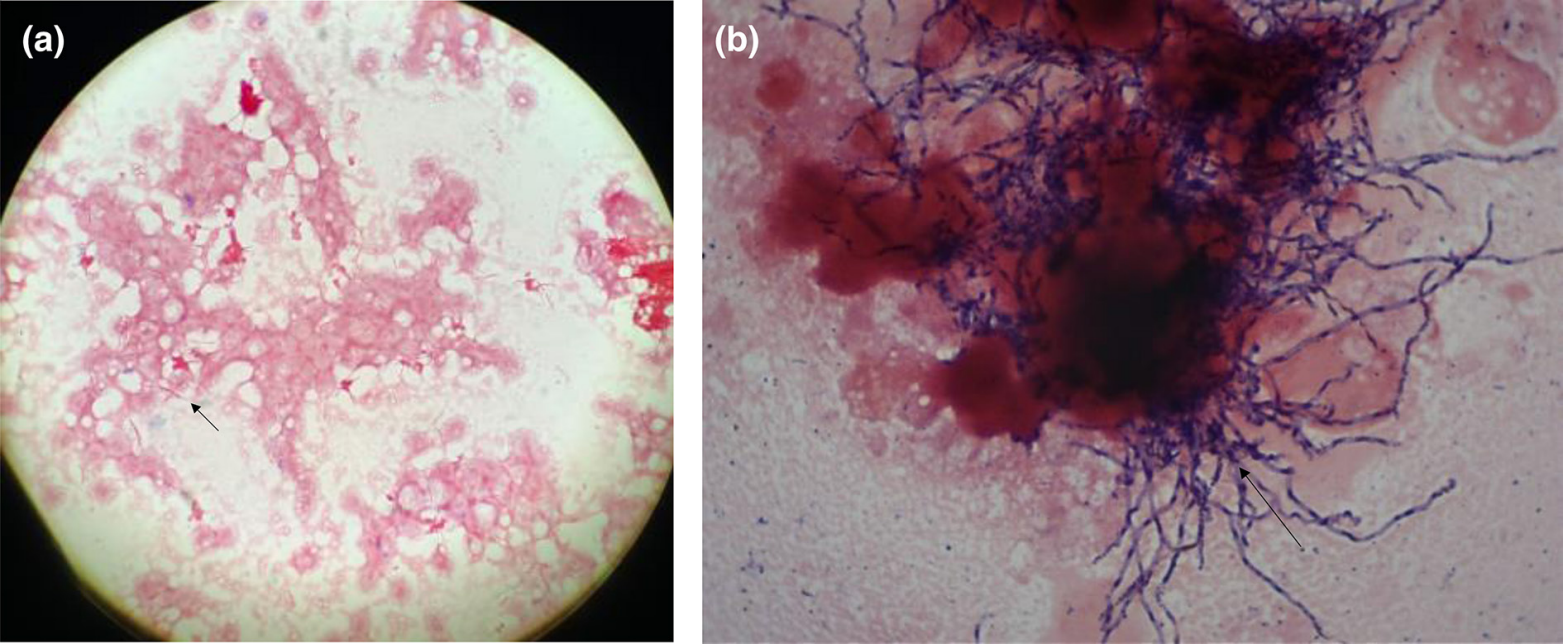
(**a**): Gram-staining suggestive of *Fusobacterium* sp. (**b**): Gram-staining suggestive of *Actinomyces* sp.

Concerning the microbiology, macroscopic analysis showed the presence of sulphur granules viewable on the collection tube, suggesting the presence of a bacterium of the actinomycetal family (Fig. 4). In this context, after crushing these and taking a smear for direct examination at ×1000 magnification after Gram-staining, the sulphur granules proved to contain branched Gram-positive bacilli microscopically similar to *Actinomyces* sp. Microscopic analysis of the abscess drainage fluid at ×1000 magnification following Gram-staining revealed moderately numerous leukocytes (6–30/field) and a polymicrobial population. Rare spindle-shaped Gram-negative bacilli suggestive of *Fusobacterium* sp. (Fig. 5a), as well as rare Gram-positive cocci and fine pleomorphic, branched Gram-positive bacilli consistent with *Nocardia* sp. or *Actinomyces* sp. were observed as in crushed granules (Fig. 5b). The anaerobic culture performed at 35–37 °C, confirmed after 2 days of incubation the presence of *F. nucleatum* in moderate quantity and rare *Propionibacterium acnes* (likely skin contaminant). After 3 days of incubation, aerobic and anaerobic culture confirmed the presence of moderate quantity of *A. israelii *. The aerobic culture also demonstrated the presence of a rare quantity of two other bacteria with unknown clinical significance: *Micrococcus luteus* (likely skin contaminants) and *Janibacter indicus* (likely environmental contaminant). All bacterial identifications were performed by Matrix Assisted Laser Desorption Ionization - Time of Flight, mass spectrometry. An antibiotic susceptibility testing for *A. israelii* was performed, using the European Committee on Antimicrobial Susceptibility Testing (EUCAST) v.9.0 (2019) breakpoints for Gram-positive anaerobic bacteria for the interpretation. It demonstrated the susceptibility of *A. israelii* to penicillin, piperacillin/tazobactam and clindamycin. Otherwise, no antibiogram could be performed for *F. nucleatum* because of growth failure after subculture.

**Fig. 6.**
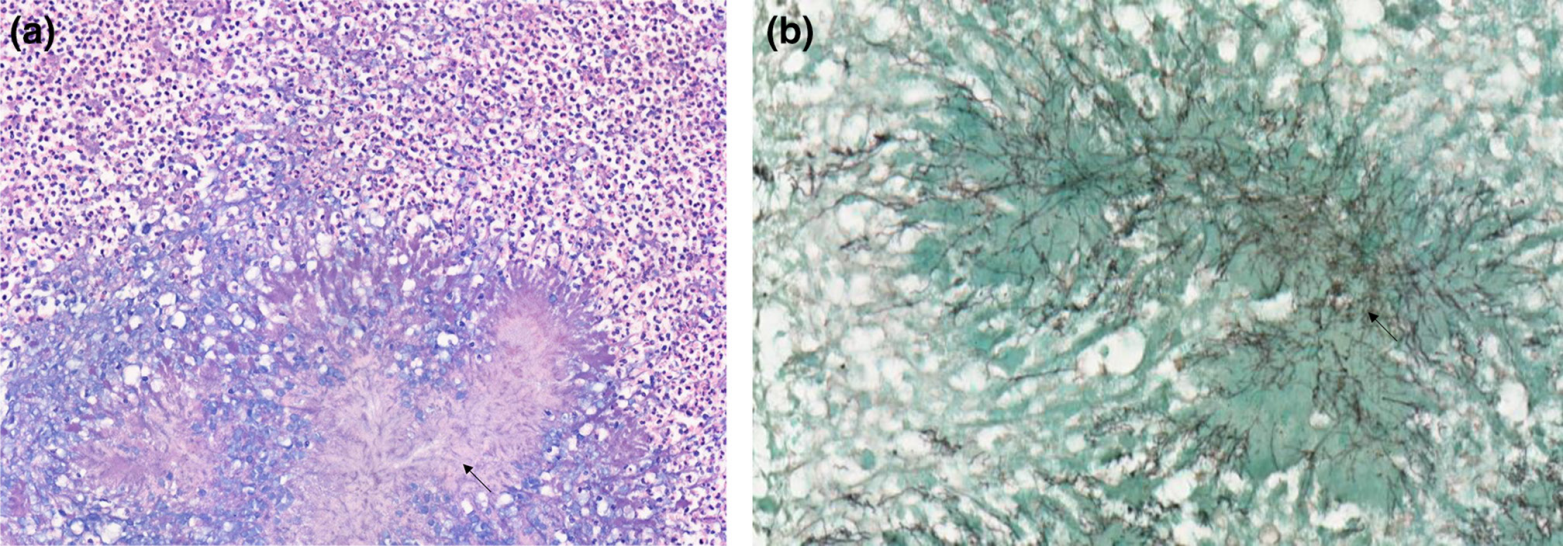
(**a**): Hematoxylin and Eosin staining suggestive of *Actinomyces* sp. (**b**): Grocott staining suggestive of *Actinomyces* sp.

Pathologically, the samples taken showed fragments of brain parenchyma with numerous necrotic, abscessed cavities, consisting mainly of neutrophils and a few rare lymphocytes. Rare colonies of *Actinomyces* sp. were found in the centre of some cavities (Fig. 6). The rest of the parenchyma showed a glial reaction. The leptomeninges and vessels passing through them were surrounded by a lymphocytic reaction. No significant atypia was observed. The immunohistochemically examinations performed were reassuring and ruled out a malignant process.

Based on these bacteriological findings, vancomycin was stopped. Although the necessary additional dental examinations could not be carried out, oral examination and CT scan of the face showed the most likely hypothesis to be periodontitis. The hypothesis of an ear, nose, or throat infection with locoregional extension causing the brain abscess was maintained as the most likely one.

### Follow-up and outcome

**Fig. 7.**
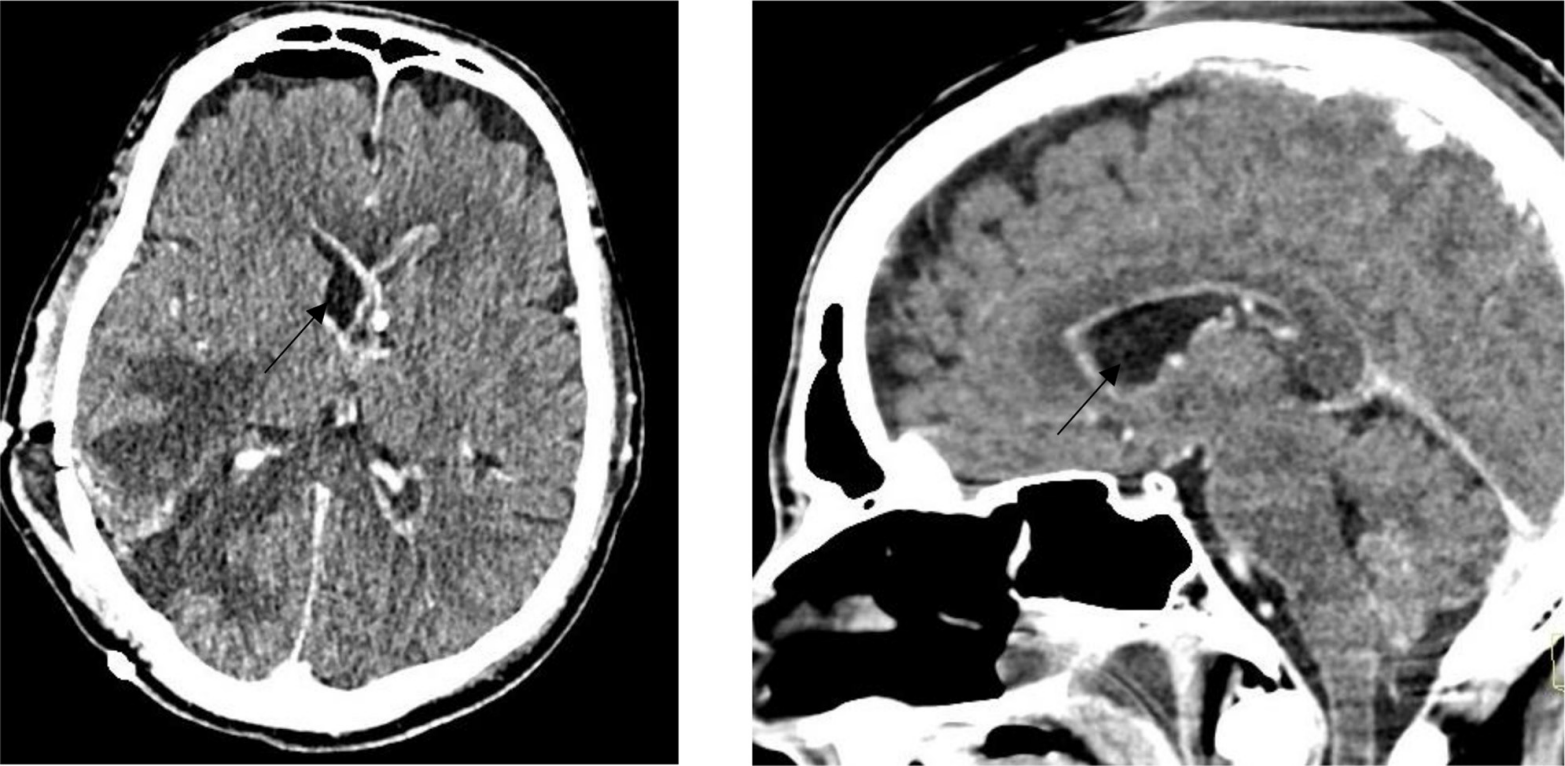
Post-operative brain contrast-enhanced computed tomography demonstrating the ventriculitis.

During the hospitalization in intensive care, a massive ventriculitis progressively appeared and was refractory to attempts of external shunting and antibiotic therapy (Fig. 7). Due to the lack of recovery of consciousness despite the gradual cessation of sedation and the prognosis, comfort care was instituted in a collegial manner, leading inevitably to the patient’s death a few hours later.

## Discussion

With more than 1200 bacterial types, the oral cavity is a major entry point for various pathogens. Several studies have demonstrated the involvement of bacteria of the oropharyngeal flora in the pathophysiology of many systemic, cardiovascular and cerebral diseases [3, 8]. Dental infection, and particularly periodontitis and dental caries are a rare but possible starting point for brain abscesses through haematogenous spread [1–6, 8, 11–14].

Bacterial infection are at least involved in 85 % of spontaneous cases of brain abscesses, the two-thirds are caused by dental and oropharyngeal microbiota [15]. Between 30–60 % of brain abscesses are polymicrobial, making the identification of the primary infection cause difficult [7]. Periodontitis and caries leading to periapical involvement are the main dental diseases that can lead to CNS infection [6]. In this context, the identification of the causal microorganisms becomes an essential element.

Such as in this case report, the fragility of some bacteria, particularly anaerobic microorganisms, may prevent the performance of an antibiogram [8]. The strict anaerobic nature of some bacteria requires the most efficient coordination between the laboratory and clinicians to minimize the delay in sample delivery. In oral CNS infections, aerobic bacteria promote the growth of the anaerobic via overconsumption of oxygen at the infection site. In this context, anaerobic bacteria are three times more involved than the aerobic in this pathology [6]. The literature describes the responsibility of the oral microbiota in brain abscesses, showing the major involvement (32 %) of viridans streptococci of the milleri family (*Streptococcus (s). anginosus, S. constellatus* and *S. intermedius *), followed *by F. nucleatum* (13.6 %), *Porphyromonas gingivalis* (6.8 %) and *Actinomyces* sp. (2 %) [9, 16].

*A. israelii *, the predominant bacteria isolated in the brain abscess, is a bacterium belonging to the *Actinomyces* group. Filamentous prokaryotic microorganisms, Gram-positive bacilli, branching filamentous rods (except *A. meyeri *), non-acid-fast, anaerobic or microaerophilic, *Actinomyces* are present in the commensal flora of the oropharynx, gastrointestinal and urogenital tract [10, 17]. *Actinomyces* take advantage of the phenomenon of co-aggregation. Indeed, they profit of the hypoxia caused by aerobic bacteria, the toxins and enzymes that the latter secrete, to potentially cause actinomycosis, which is a rare, suppurative, granulomatous endogenous infection [6, 18]. Unlike the usual bacterial infections, actinomycosis penetrates the anatomical planes to become a lobular ‘pseudotumour’ responsible for a chronic granulomatous infection characterized by the formation, in 75 % of cases, of tiny clusters (0.1 to 1 mm) called sulphur granules [10]. Observed in our patient and strongly suggestive of actinomycosis, sulphur granules owe their name to their yellowish colour and allow the microorganisms to resist phagocytosis [10, 19, 20]. *Actinomyces* grow on blood-enriched agar medium with a 2–4 days incubation at 35–37 °C under anaerobic conditions [21]. In agreement with the antibiogram performed in our laboratory, the literature describes a sensitivity of all *Actinomyces* sp. to beta-lactam antibiotics, making this class of antibiotics the first line of treatment for actinomycosis, while metronidazole is considered inactive [10, 22].

*F. nucleatum *, a non-spore-forming, non-motile Gram-negative bacillus, is the main pathogen of the *Bacteroidaceae* family. As a strict anaerobe, which can be found in the oral cavity, respiratory, gastrointestinal and genitourinary tracts, *F. nucleatum* is able to bridge the gap between oral colonisers and dental plaque bacteria. It then facilitates the formation of a biofilm allowing the coexistence of bacteria with conflicting environmental requirements [23, 24]. *F. nucleatum* is strongly implicated in periodontal disease [25] through lectin-like interactions, and has a strong ability to invade gingival epithelial cells leading to an exacerbation of neutrophilic activity, resulting in tissue damage, which can lead to abscess formation [26]. *Fusobacterium* can evade the immune system due to their ability to bind to the constant fragment of immunoglobulins suggesting that dissemination can occur during transient bacteremia [27]. In correlation with our patient, rare cases of *F. nucleatum* associating brain abscesses with a periodontal origin via haematogenous spread have been described in the literature [25, 28]. At the therapeutic level, azithromycin, metronidazole, clindamycin and colistin are considered active. Reduced susceptibility to neomycin, erythromycin, amoxicillin and ampicillin has been observed [26, 29]. The secretion of β-lactamase or penicillinase by *F. nucleatum* has been described [26, 30].

Considered a public health problem, periodontitis in our case remains uncertain. The presence of oral germs found on the samples and frequently associated with periodontitis; the loss of alveolar bone estimated radiographically, and the significant loosening of the teeth observed on dental examination; are elements in favour but insufficient for a diagnosis of definite periodontitis [31]. Unfortunately, due to the critical condition of the patient requiring intubation as well as the lack of contribution to the immediate management of the patient, a more thorough diagnosis of the patient’s oral pathology was not performed before the patient’s death.

In the absence of international guidelines, recommendations for antibiotic therapy were published on behalf of the British Society of Antimicrobial Chemotherapy in 2000 [32]. Thus, empirically, after removal and/or aspiration of the brain abscess, the choice and duration of antibiotic therapy depends on the initial site of infection and the location of the abscess. In polymicrobial abscesses, the use of ceftriaxone/metronidazole dual therapy covers Gram-positive and Gram-negative bacteria as well as anaerobes and appears to be effective [28, 32, 33]. Although this dual therapy seems to be a good therapeutic approach for brain abscesses with a suspected oral aetiology, bacteriological documentation is of great importance for the adaptation of antibiotic therapy towards less broad spectra, or in the case of the demonstration of resistance mechanisms.

In conclusion, periodontal abscesses, and other infections of dental origin in rare cases, can be responsible for brain abscesses. The clinical examination of patients with neurological disorders that can be explained by the presence of a brain abscess should include a systematic dental inspection to check for periapical abscesses. Given the difficulty of growth and the anaerobic nature of the bacteria originating from the oral microbiota and involved in brain abscess, special attention should be paid to the rapid transport of the sample to the laboratory and to the prolonged incubation of the culture. As demonstrated in our case, the macroscopic examination of the specimens can be as important as the microscopic examination for the orientation of further investigations in the laboratory. Based on the microbiological findings, surgical drainage and empirical dual therapy with ceftriaxone/ metronidazole appears to be the optimal treatment strategy. However, despite the existence of multiple effective antibiotic therapies, brain abscesses can be fatal as demonstrated in our case. *F. nucleatum* plays a very important role in the co-aggregation of bacteria in the oral microbiota. In this context, empirical coverage of this pathogen in cases of oral brain abscesses is therefore crucial. This underlines the importance of following the international recommendations for empirical treatment of abscesses according to their suspected origin.
